# Animal Models Are Valid to Uncover Disease Mechanisms

**DOI:** 10.1371/journal.pgen.1006013

**Published:** 2016-05-26

**Authors:** Wolfgang Wurst

**Affiliations:** 1 Helmholtz Zentrum München, German Research Center for Environmental Health Institute of Developmental Genetics, Munich, Germany; 2 Technische Universität München-Weihenstephan, Chair of Developmental Genetics c/o Helmholtz Zentrum München, Munich, Germany; 3 German Center for Neurodegenerative Diseases (DZNE), Munich, Germany; 4 Munich Cluster for Systems Neurology (SyNergy) Ludwig-Maximilians-Universität München, Munich, Germany; University College London, UNITED KINGDOM

## Background

Autism spectrum disorder (ASD) is a complex, neuropsychiatric disease, characterized mainly by social interaction deficits, language impairments, and repetitive, stereotyped behaviors already apparent in early childhood. The incidence of ASD is currently about one in 100 newborns with an increasing frequency. ASD is a very heterogeneous disease with a high degree of phenotypic and genotypic variability. More than 100 genes and genomic regions have been associated with ASD, each associated only with a small subset of patients. ASD is primarily diagnosed based on the occurrence of two behaviors: (i) stereotyped and repetitive patterns of behavior and restricted interest and (ii) impaired social interaction and communication. However, there are additional phenotypic alterations observed, such as mental retardation, anxiety, attention deficit hyperactive disorder (ADHD), and epilepsy. The genetic complexity and the behavioral variations pose a major obstacle in modelling ASD in mice. Mice are still necessary in order to unravel underlying molecular mechanisms. The insights into these molecular mechanisms will lead to better treatment regimens. Thus, animal models are valuable and urgently needed. However, to achieve this goal, at least two major difficulties have to be addressed: (i) the often imprecise modelling of the human genomic mutation and, foremost, (ii) the neglect of the effects that genetic background has on the manifestations of symptoms. The latter is of utmost importance for the field of mouse models in ASD since it is known that behavior, which is up to now the only diagnostic criteria for ASD (see above), is strongly dependent on the genetic background [1,2].

## What Is New?

In ASD, copy number variations (CNVs) are known to be associated with the disease. While modelling the effect of disease-associated gene mutations, i.e., loss-of- function (knock-out), is currently quite straightforward concerning construct validity, modelling the effect of CNVs affecting a large genomic region is still a challenge. The CNV addressed in the report of Arbogast et al. [3] is located at chromosome 16p11.2, encompassing 600 kb between BP4–BP5 breakpoint (BP) deletions and duplications, and accounts for approximately 1% of all ASD cases [4]. The affected genomic region accommodates 28 protein-coding genes, most of which are expressed in the brain. Adding to already existing mouse models of this CNV [5,6], Arbogast et al. have modelled the deletion and duplications in a highly precise manner: 1. They modelled the CNV at precise orthologous positions to include all protein-coding genes so that no additional genomic elements within the syntenic region might interfere with the symptomatic outcome. 2. They generated both deletion and duplication of the precise syntenic region in the mouse in order to assess the copy number effect of the mutation. Thus, they have convincingly addressed the first criticism concerning appropriate mouse models in ASD.

Arbogast et al. carried out the initial behavioral analysis of the mice carrying either the duplication or the deletion of this genomic region on a pure C57BL/6N background. Mice carrying the deletion of the genomic region displayed hyperactivity, repetitive behaviors, and memory deficits reminiscent of ASD symptoms. In contrast, the mice carrying a duplication of the region behaved mostly the opposite. In order to prove the effect of the copy number of this region, the authors generated pseudo-disomic mice by crossing the deletion mutant with the duplication mutant. Indeed, most of the observed phenotypes were restored to normal levels in these mice, proving the implication of dosage-sensitive genes possibly influencing the above behaviors. This hypothesis is substantiated by the transcriptome analysis performed in this study, showing that the majority of genes in these genomic regions exhibit mRNA levels proportional to the CNV copy number.

**Fig 1:**
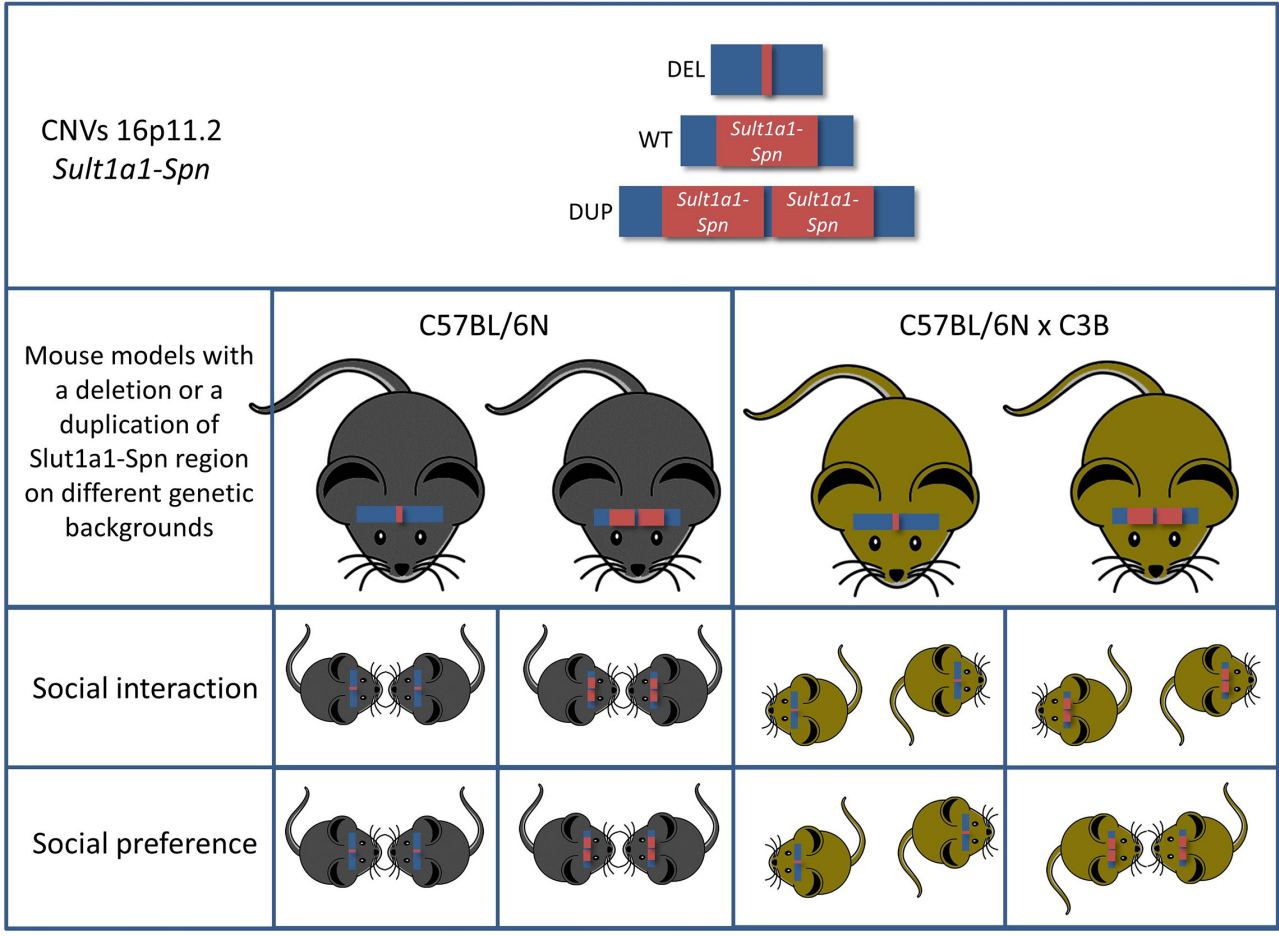
Mice carrying a deletion syntenic to the human chromosome 16p11.2 (Sult1a1-Spn) exhibit multiple behavioral phenotypes associated with ASD. Intriguingly, two of the key behavioral phenotypes (social interaction and social preference) of ASD are highly genetic-background–dependent.

All these findings are in good agreement with earlier mouse models modelling the 16p11.2 BP4-BP5 CNVs. However, the authors carried the analysis further by addressing the effect of the genetic background, thus addressing the second important criticism concerning modelling ASD in mice. They found that the genetic background has a profound effect on the behavioral outcome. The genetic background effect was obvious in very evident phenotypes—such as rates of neonatal lethality in the deletion mutants—but, most importantly, mice with a C57BL/6NxC3B hybrid background displayed a social interaction phenotype not yet reported in any mouse model for this region (Fig 1). This is most likely due to the fact that in C57BL/6N wild-type mice social interaction phenotypes are hard to detect. Indeed, Arbogast et al. showed that the level of social interaction of mice of this inbred strain is low when compared to the interaction level of mice on a C57BL/6NxC3B hybrid background. Thus, it is of the highest importance to include the effects of the genetic background in the analysis of behavioral phenotypes, both at the level of the analysis and at the level of the experimental design.

Another aspect of this study that jumps out at the reader immediately is the obvious discrepancy concerning the metabolic defects between mice and humans carrying the 16p11.2 BP4-BP5 rearrangements. Even though the mice show a phenotype in this respect, it is the opposite of what is observed in humans; i.e., deletions lead to weight loss in mice, whereas humans with deletions in this region exhibit obesity. Interestingly, this phenotype was not altered by the genetic background indicating that the effects on metabolic function(s) are robust. A similar discrepancy between human and mouse phenotypes was observed concerning skull morphology. These phenotypic discrepancies might reflect the occurrence of small genomic changes during evolution affecting the regions, i.e., where the BP take place, resulting in altered interaction of elements located outside the BP with genes within. This hypothesis again pushes the first criticism (that is the precise modelling of CNVs in ASD) to the next level, in which the surrounding regions of the BP also need to be considered.

## The Next Levels

With regard to modelling of ASD in mice, the paper by Arbogast et al. made a clear statement concerning the importance of the genetic background for detecting behavioral ASD-related phenotypes as well as for the importance of the introduction of the precise mutation. The latter will be improved due to the discovery of the CRISPR/Cas9 technology, which allows fast generation of nested, reciprocal gene deletions and duplications. Thus, we will be able to narrow down the genes responsible for the different phenotypic alterations associated with chromosomal 16p11.2 deletions and duplications followed by gene-specific loss- and gain-of-function studies [7–9]. Furthermore, the dynamic alterations of transcriptional regulation and protein interaction networks of the genes located within the BPs during critical periods of development will enable us to unravel the molecular networks and possible mechanisms underlying the various symptoms [10]. Last but not least, employing an array of brain circuit visualization and interrogation tools such as CLARITY, viral tracing, and optogenetics in combination with the various deletion and gene knockout models will allow us to determine altered neuronal subtypes, their projections, biophysical properties, as well as activity patterns [11], giving insights at the systemic level of the disease.

There are still additional issues to be addressed in the field of modelling ASD: the multifactorial etiology of ASD and the evolutionary (species-specific) aspects. Whereas the first might still be achieved, e.g., evaluation of environmental impact, the latter naturally will preclude a 1:1 modelling of the disease in animals. However, if we curtail our expectations of animal models in respect to reflecting the full-blown pathology of the diseases by mirroring exactly all phenotypes—specifically, behavior—we will start to re-appreciate their usefulness for our understanding of the molecular mechanisms of the disease process. Taken together, the findings and approaches highlighted in this paper—by working towards better genetic modelling strategies, taking into account the effects of genetic background, and, specifically, combining phenotypic analysis with molecular analysis—are pointing the way towards understanding disease-causing molecular and cellular mechanisms, which are the ultimate target for disease-modifying therapies.

## References

[1] Jaramillo TC, Liu S, Pettersen A, Birnbaum SG, Powell CM (2014) Autism-related neuroligin-3 mutation alters social behavior and spatial learning. Autism Res 7: 264–272. 10.1002/aur.1362 24619977 PMC3989414

[2] Keane TM, Goodstadt L, Danecek P, White MA, Wong K, et al (2011) Mouse genomic variation and its effect on phenotypes and gene regulation. Nature 477: 289–294. 10.1038/nature10413 21921910 PMC3276836

[3] Arbogast T, Ouagazzal A, Chevalier C, Kopanitsa M, Afinowi N, et al (2016) Reciprocal effects on neurocognitive and metabolic phenotypes in mouse models of 16p11.2 deletion and duplicaton syndroms. PLoS Genet 12(2): e1005709 10.1371/journal.pgen.1005709 26872257 PMC4752317

[4] Walsh KM, Bracken MB (2011) Copy number variation in the dosage-sensitive 16p11.2 interval accounts for only a small proportion of autism incidence: a systematic review and meta-analysis. Genet Med 13: 377–384. 10.1097/GIM.0b013e3182076c0c 21289514

[5] Horev G, Ellegood J, Lerch JP, Son YE, Muthuswamy L, et al (2011) Dosage-dependent phenotypes in models of 16p11.2 lesions found in autism. Proc Natl Acad Sci U S A 108: 17076–17081. 10.1073/pnas.1114042108 21969575 PMC3193230

[6] Portmann T, Yang M, Mao R, Panagiotakos G, Ellegood J, et al (2014) Behavioral abnormalities and circuit defects in the basal ganglia of a mouse model of 16p11.2 deletion syndrome. Cell Rep 7: 1077–1092. 10.1016/j.celrep.2014.03.036 24794428 PMC4251471

[7] Chavez A, Scheiman J, Vora S, Pruitt BW, Tuttle M, et al (2015) Highly efficient Cas9-mediated transcriptional programming. Nat Methods 12: 326–328. 10.1038/nmeth.3312 25730490 PMC4393883

[8] Kraft K, Geuer S, Will AJ, Chan WL, Paliou C, et al (2015) Deletions, Inversions, Duplications: Engineering of Structural Variants using CRISPR/Cas in Mice. Cell Rep. 10.1016/j.celrep.2015.01.016 25660031

[9] Yang H, Wang H, Shivalila CS, Cheng AW, Shi L, et al (2013) One-step generation of mice carrying reporter and conditional alleles by CRISPR/Cas-mediated genome engineering. Cell 154: 1370–1379. 10.1016/j.cell.2013.08.022 23992847 PMC3961003

[10] Lin GN, Corominas R, Lemmens I, Yang X, Tavernier J, et al (2015) Spatiotemporal 16p11.2 protein network implicates cortical late mid-fetal brain development and KCTD13-Cul3-RhoA pathway in psychiatric diseases. Neuron 85: 742–754. 10.1016/j.neuron.2015.01.010 25695269 PMC4335356

[11] Lerner TN, Shilyansky C, Davidson TJ, Evans KE, Beier KT, et al (2015) Intact-Brain Analyses Reveal Distinct Information Carried by SNc Dopamine Subcircuits. Cell 162: 635–647. 10.1016/j.cell.2015.07.014 26232229 PMC4790813

